# *TP53* and *PTEN* mutations were shared in concurrent germ cell tumor and acute megakaryoblastic leukemia

**DOI:** 10.1186/s12885-019-6497-0

**Published:** 2020-01-02

**Authors:** Keiichi Akizuki, Masaaki Sekine, Yasunori Kogure, Takuro Kameda, Kotaro Shide, Junji Koya, Ayako Kamiunten, Yoko Kubuki, Yuki Tahira, Tomonori Hidaka, Takumi Kiwaki, Hiroyuki Tanaka, Yuichiro Sato, Hiroaki Kataoka, Keisuke Kataoka, Kazuya Shimoda

**Affiliations:** 1 Department of Gastroenterology and Hematology, Faculty of Medicine, University of Miyazaki, 5200 Kihara, Kiyotake, Miyazaki, 889-1692 Japan; 2 Division of Molecular Oncology, National Cancer Center Research Institute, Tokyo, Japan; 3 Department of Pathology, Faculty of Medicine, Section of Oncopathology and Regenerative Biology, University of Miyazaki, 5200 Kihara, Kiyotake, Miyazaki, 889-1692 Japan; 4 Department of Diagnostic Pathology, Department of Pathology, Faculty of Medicine, University of Miyazaki, 5200 Kihara, Kiyotake, Miyazaki, 889-1692 Japan

**Keywords:** Acute myeloid leukemia, Germ cell tumor, *TP53*, *PTEN*

## Abstract

**Background:**

The occurrence of a mediastinal germ cell tumor (GCT) and hematological malignancy in the same patient is very rare. Due to its rarity, there have been only two reports of the concurrent cases undergoing detailed genetic analysis with whole-exome sequencing (WES), and the possible clonal relationship between the both tumors remained not fully elucidated.

**Methods:**

We performed whole-exome sequencing analysis of mediastinal GCT and acute myeloid leukemia (AML) samples obtained from one young Japanese male adult patient with concurrent both tumors, and investigated the possible clonal relationship between them.

**Results:**

Sixteen somatic mutations were detected in the mediastinal GCT sample and 18 somatic mutations in the AML sample. Mutations in nine genes, including *TP53* and *PTEN* both known as tumor suppressor genes, were shared in both tumors.

**Conclusions:**

All in our case and in the previous two cases with concurrent mediastinal GCT and AML undergoing with whole-exome sequencing analysis, *TP53* and *PTEN* mutations were commonly shared in both tumors. These data not only suggest that these tumors share a common founding clone, but also indicate that associated mediastinal GCT and AML harboring *TP53* and *PTEN* mutations represent a unique biological entity.

## Background

Germ cell tumors (GCTs) are the most common malignant tumors in adolescent males. Approximately, 2–5% of GCTs arise at extragonadal sites [1]. Among them, mediastinal GCTs (mGCTs) predominantly occur within the anterior mediastinum. Though mGCTs have different clinical characteristics from testicular GCTs, those were thought to be derived from gonadal lesions as there was no cytogenetic difference between them [2]. Since 1985, the unique and rare associations between hematological malignancies (HMs) and mGCTs were reported in approximately 60 cases [3, 4]. In most cases, the involved GCT was non-seminomatous and mediastinal, and the HM was acute myeloid leukemia (AML), frequently acute megakaryoblastic leukemia (AMKL) under the WHO 2017 classification, corresponding to AML M7 under the former French-American-British classification. The associations with myelodysplastic syndrome (MDS), myelomonocytic leukemia, and essential thrombocythemia have also been reported [4, 5]. The interval between the onset of mGCTs and that of HMs is occasionally < 6 months, and the synchronous presentation of the two diseases is sometimes observed. HMs associated with mGCTs should be separated from therapy-related secondary AML or MDS, which typically develop at least a year following exposure to cytotoxic drugs administered for GCT treatment. The association of HMs with mGCTs is extremely rare. In a large, international, multicenter database study of 635 extragonadal GCT patients, HMs were observed in 17 extragonadal GCTs [5]. All cases were mGCT cases and considering that there were 287 mGCT cases in total, the incidence rate of concurrent mGCT and HM in this group was 6%. The frequent presence of isochromosome 12p in AML samples from these patients strongly suggested that the HMs and mGCTs might arise from common progenitor cells, because isochromosome 12p is the most common chromosomal abnormality in GCTs, but is exceptionally rare in AML without mGCT association [5–8]. Recently, two patients were reported to have *TP53* and *PTEN* mutations in concurrent AML and mGCT in each patient from two independent reports [9, 10]. One of them was Caucasian and the other was not referred for its ethnicity. This discovery not only strengthened the concept of the common progenitor cells, but also provided insights into the molecular aspects of this unique and rare association [9, 10]. Herein, we report a third case of the concurrent occurrence of mediastinal GCT and AMKL, in which we performed whole-exome sequencing (WES) analysis of both tumors and investigated the possible clonal relationship between them.

## Methods

### Sample collection

This study was approved by the Research Ethics Committee of the Faculty of Medicine, University of Miyazaki. GCT samples (the left cervical mass) and AML samples (bone marrow) were obtained from the patient with written informed consent.

### Cytogenetic analysis

Cytogenetic analyses were performed by G-banding on GCT and AML samples, and interphase fluorescence in situ hybridization (FISH) on the frozen stocked GCT sample. In FISH analysis, human 6p22/6q22 probe, 8 centromere/21q22 probe, and 12p12/12q14 probe (Chromosome Science Labo Inc., Sapporo, Japan) were used. Frozen cells were thawed and washed by PBS. After treatment of 0.075 mol/L KCL for 20 min at room temperature. Cells were fixed 3 times with methanol:acetic acid = 3:1 and fixed cells were spread on slides. Probes were applied to the cell spreads, covered with cover slips and simultaneously denatured at 70 °C for 5 min and hybridized overnight at 37 °C. The hybridized slide was washed and counterstained with 4′,6-diamidino-2-phenylindole and mounted in anti-fade solution. Separate fluorochrome images were captured using a Leica DC 350FX cooled CCD camera (Leica, Wetzlar, Germany) mounted on a Leica DMRA2 microscope using Leica CW4000 FISH software. The images were analyzed using Leica CW4000 karyo software (Leica).

### DNA extraction and WES analysis

Genomic DNA from GCT and AML samples was extracted using the QIAamp DNA Mini kit. WES analysis of GCT and AML samples were performed using the patient’s buccal mucosa as a germline control, as previously described [11]. SureSelect Human All Exon v6 kits (Agilent Technologies) were used for exome capture according to the manufacturer’s instructions. Sequencing data were generated using the Illumina NextSeq 500 platform with a standard 150-bp paired-end read protocol, as previously described [11]. Sequence alignment and mutation calling were performed using the Genomon pipeline (https://github.com/Genomon-Project), as previously described. Putative somatic mutations with (i) Fisher’s exact *P* value < 0.01; (ii) > 2 variant reads in tumor; (iii) allele frequency in tumor ≥0.035; and (iv) allele frequency in germline < 0.035 were adopted and filtered by excluding (a) synonymous single nucleotide variants (SNVs); (b) variants only present in unidirectional reads; and (c) variants occurring in repetitive genomic regions. These candidate mutations were further filtered by removing known variants listed in NCBI dbSNP build 131, the 1000 Genomes Project (October 2014 release), National Heart, Lung, and Blood Institute (NHLBI) Exome Sequencing Project (ESP) 6500, and the Human Genome Variation Database, unless they were listed in the COSMIC database (v70). Finally, all detected mutations were manually checked by Integrative Genomics Viewer (IGV) and their allele frequencies were calculated using pysam’s pileup function (version 0.14.1).

## Results

### Clinical and pathological findings

**Fig. 1:**
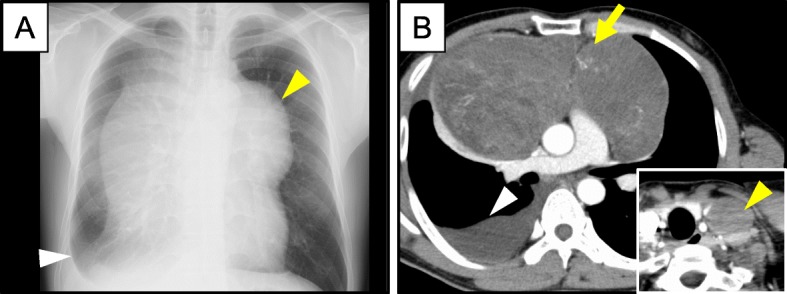
Chest X-ray and CT scan. **a** Chest X-ray reveals a well-circumscribed bilateral hilar mass approximately 20.5 cm in maximum dimension (yellow arrowhead), and dullness of the right costal pleural angle (white arrowhead). **b** CT scan reveals a 19.5 cm × 10.8 cm heterogeneously enhancing anterior mediastinal mass (yellow arrow) and a 4.3 cm left cervical mass (yellow arrowhead), and right pleural effusion (white arrowhead)

**Fig. 2:**
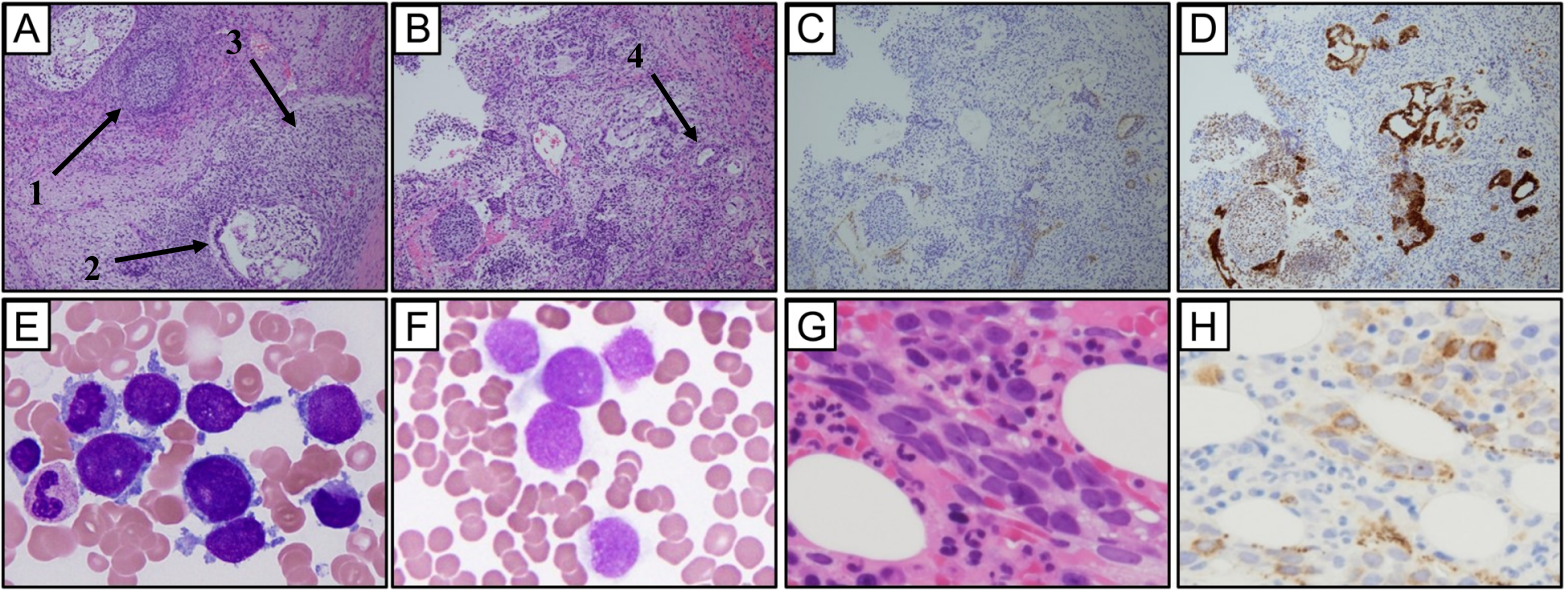
Histopathology of the left cervical mass shows features of non-seminomatous germ cell tumor. Cytology and histopathology of bone marrow (BM) shows features of acute megakaryoblastic leukemia. **a** An open biopsy sample of the left cervical mass shows immature teratoma (arrow 1), columnar epithelium (arrow 2), and immature mesenchymal components (arrow 3) [hematoxylin and eosin staining (H.E.)., 200×] and (**b**) yolk sac tumor-like components (arrow 4) (H.E., 200×). **c** Immunohistochemically, the yolk sac tumor-like components are weakly positive for alpha-fetoprotein (AFP) (100×) and (**d**) strongly positive for Glypican-3 (100×). **e** BM smear reveals many large blasts with nuclear and cytoplasmic blebs (Giemsa staining, 1000×). **f** Blast cells are negative for myeloperoxidase. **g** BM biopsy shows increased blast cells (H.E., 400×). **h** Immunohistochemically, blast cells show strong cytoplasmic positivity for von Willebrand factor (400×)

The patient was a 37-year-old Japanese, previously healthy male who presented with a dry cough. He first visited his family doctor and was pointed out to have a 5-cm diameter left cervical tumor, following which, he was referred to our hospital. Examination revealed tachycardia (107/min) and elastic hard left cervical mass with a 5 cm diameter. A chest X-ray revealed a well-circumscribed bilateral hilar mass with a maximum dimension of 20.5 cm, and dullness of the right costal pleural angle (Fig. 1a). Peripheral blood examination showed the following: Hb level 16.2 g/dL; leucocyte count 9.9 × 10^9^/L, and platelet count 293 × 10^9^/L. Serum alpha-fetoprotein (AFP) (normal range: 0–8.5 ng/mL), beta-human chorionic gonadotropin (βhCG) (normal range: 0–4 mIU/mL), and lactate dehydrogenase levels (normal range: 119–213 IU/L) were 1921 ng/mL, 511 mIU/mL, and 390 IU/L, respectively. A computed tomography (CT) scan revealed a 19.5 cm × 10.8 cm heterogeneously enhancing anterior mediastinal mass and a 4.3 cm left cervical mass (Fig. 1b). A surgical biopsy of the left cervical mass showed heterogenous features including immature cartilages, immature mesenchymal cells, columnar epithelium cells, and yolk sac tumor-like components (Fig. 2a, b). Immunohistochemical staining of these tumor cells revealed immunoreactivity with AFP and Glypican-3 (Fig. 2c, d). He was diagnosed with non-seminomatous GCT, and was treated with BEP therapy (bleomycin, etoposide, and cisplatin). After starting the therapy, the serum βhCG level promptly decreased, but there was no reduction in the size of the mediastinal mass. Thrombocytopenia started 15 days after BEP therapy and persisted for 1 week. To evaluate its cause, bone marrow (BM) examination was performed. The BM aspirate showed that 74% of all nucleated cells were blasts, which were medium to large in size with round nuclei, and one to three nucleoli (Fig. 2e). These cells were negative for myeloperoxidase by immunostaining (Fig. 2f), and were positive for CD7 (79.6%), CD13 (82.6%), CD33 (81.1%), CD34 (99.1%), CD41a (99.1%), and CD117 (44.5%) by flow cytometry. BM biopsy showed hypercellular marrow, and blasts were positive for von Willebrand factor (Fig. 2g, h). The cause of cytopenia was revealed to be AMKL. Induction chemotherapy with idarubicin and cytosine arabinoside was administered for AMKL. He achieved first complete remission with enough platelet recovery. The chemotherapy for AML had no effect on the GCT, and the mediastinal mass enlarged. We therefore continued therapy for GCT with 2 courses of TIP (paclitaxel, ifosfamide, and cisplatin), 1 course of TGO (paclitaxel, gemcitabine, oxaliplatin), and finally another course of BEP therapy. These treatments did not reduce the size of the mediastinal or cervical masses. AMKL relapsed during the TIP therapy for GCT, and thrombocytopenia, which required platelet transfusion every other day, continued during the therapy. Despite these treatments, he died 6 months after his initial diagnosis.

### Cytogenetic and WES analyses

**Fig. 3:**
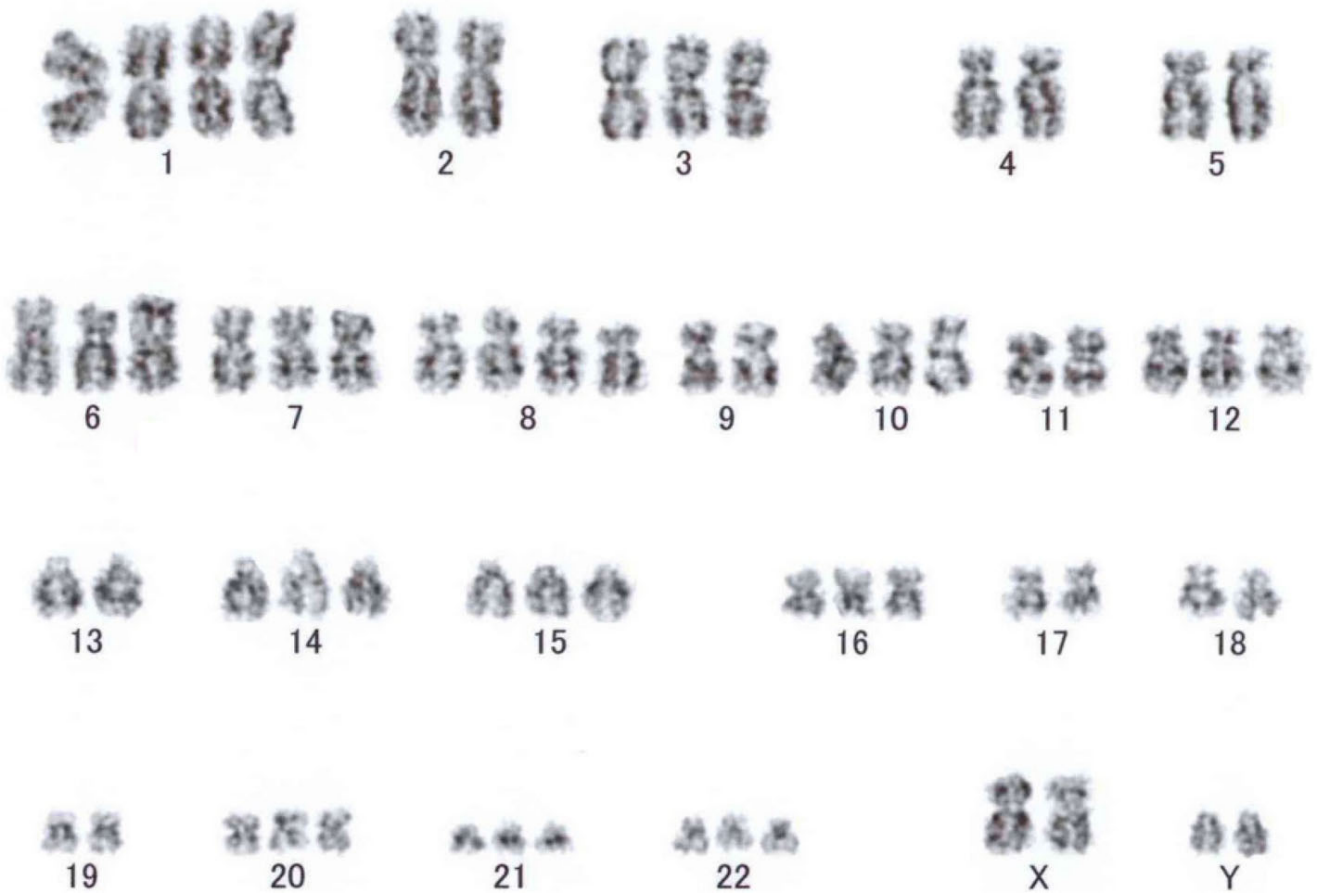
G-banding karyotype from acute myeloid leukemia sample at diagnosis

**Fig. 4:**
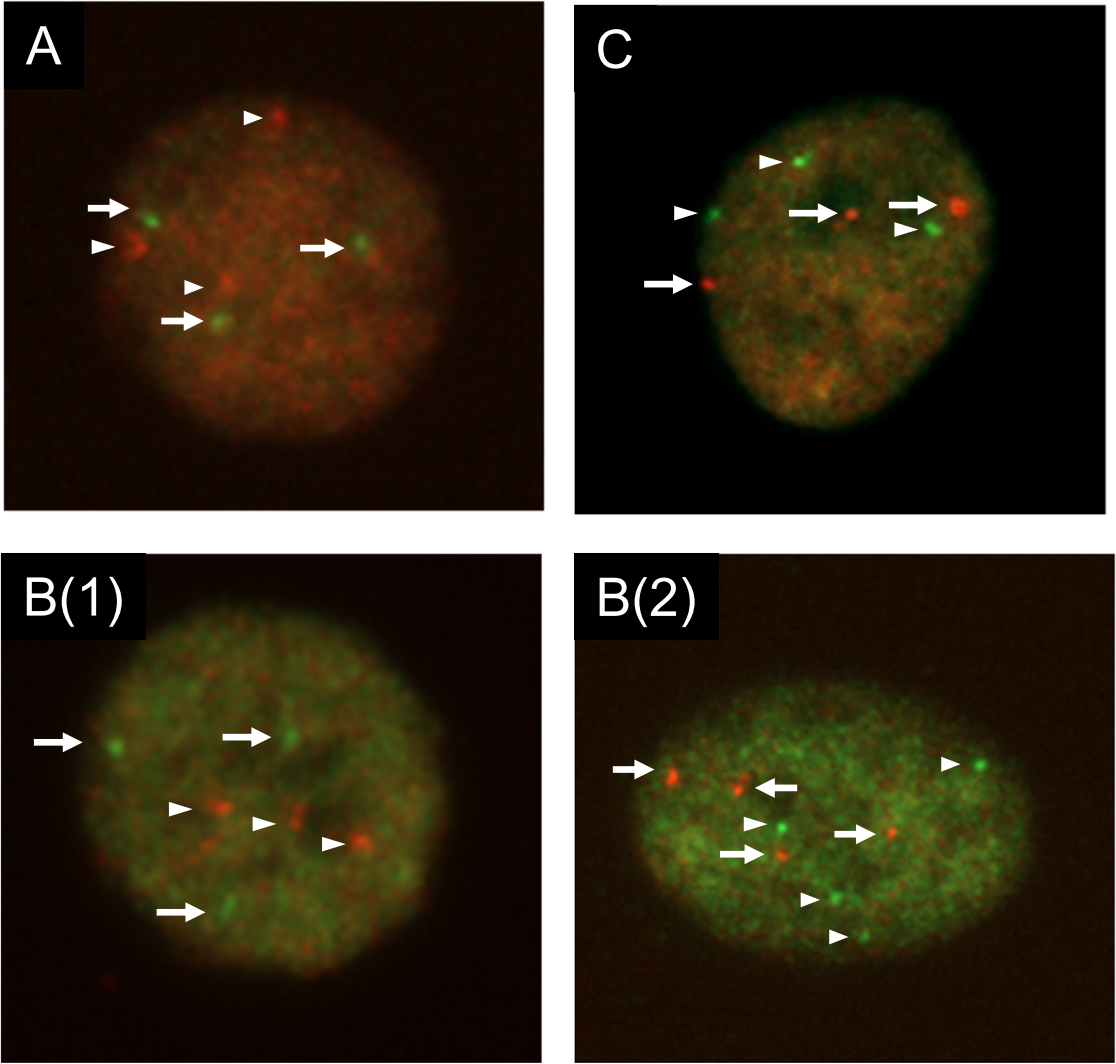
Two-color interphase FISH in germ cell tumor (GCT) sample. **a** The arrows indicate 3 green signals (6p22) and the arrowhead indicate 3 red signals (6q22) in the GCT cells. **b** The arrows indicate green signals (8 centromere probe) and the arrowhead indicate red signals (21q22). (1) trisomy 8 and trisomy 21. (2) tetrasomy 8 and tetrasomy 21. **c** The arrows indicate 3 red signals (12p12) and the arrowhead indicate 3 green signals (12q14) in the GCT cells

To clarify the possible clonal relationship between the GCT and AML, we performed cytogenetic and WES analyses of GCT and AML samples. In the cytogenetic analysis, the AML sample revealed a hyperdiploid karyotype: 63XXY,+Y,+ 1,-2,-4,-5, add(6)(p21),+ 8,-9,-11,-13,-17,-18,-19 in 4/20 metaphases and 46XY in 16/20 metaphases (Fig. 3). As no analyzable metaphases were obtained in the GCT sample, we performed two-color FISH analysis on the GCT sample using each pair probes for chromosome 6p22/6q22, 8 centromere/21q22, and 12p12/12q14. In the FISH analysis, trisomy 8, tetrasomy 8, trisomy 21, and tetrasomy 21 were detected in 11/82, 8/82, 16/84, and 15/84 mGCT cells, respectively (Fig. 4). In addition, 16/84 mGCT cells possessed three signals of both 6p22 and 6q22, and 21/85 cells showed three signals of both 12p12 and 12q14.

**Fig. 5:**
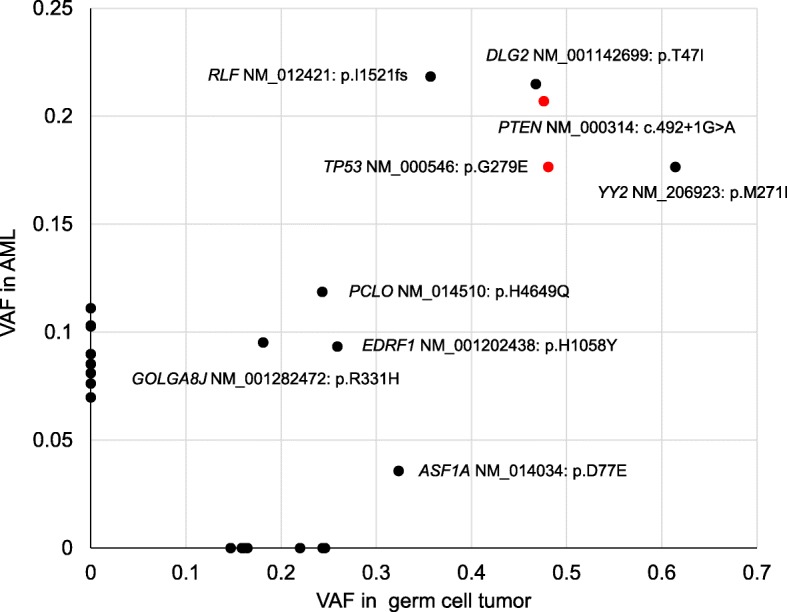
Mutated genes in germ cell tumor and acute myeloid leukemia with their variant allele frequencies. *TP53* and *PTEN* mutations are colored red. VAF, variant allele frequency; AML, acute myeloid leukemia

**Fig. 6:**
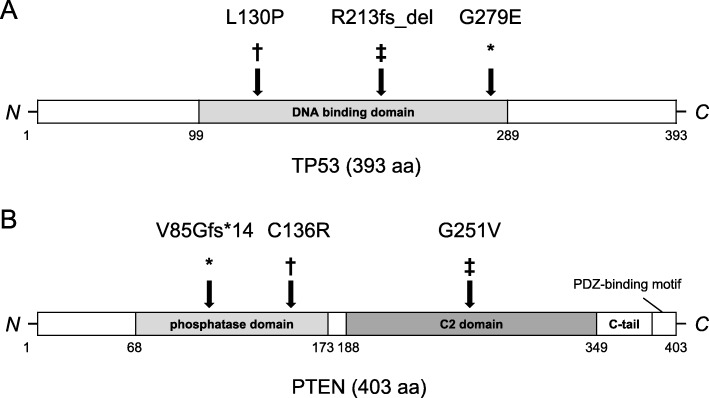
Location of *TP53* mutations (**a**) and *PTEN* mutations (**b**) in three cases including our case. * our case; ‡ Oshrine et al. [9]; † Lu et al. [10]

In the WES analysis, we detected 16 somatic mutations in the GCT sample, including 15 SNVs and one deletion, and 18 somatic mutations in the AML samples, including 17 SNVs and one deletion. Among them, mutations in 9 genes, specifically *TP53*(c.G836A)*, PTEN*(c.492 + 1G > A), *RLF*(c.4563_4567del)*, DLG2*(c.C140T)*, YY2*(c.G813A)*, PCLO*(c.T13947G)*, GOLGA8J*(c.G992A)*, EDRF1*(c.C3172T)*,* and *ASF1A*(c.T231A) were observed in both tumors and at the same nucleotide. Their detailed nucleotide changes and variant allele frequency (VAF) in each tumor are shown in Table 1. *TP53, PTEN*, *RLF, DLG2,* and *YY2* showed relatively higher VAFs than *PCLO, GOLGA8J, EDRF1*, and *ASF1A* (Table 1, Fig. 5). In our case, the *TP53* mutation (p.G279E) occurred in the DNA binding domain and the *PTEN* mutation (exon5:c.492 + 1G > A) occurred in the splicing donor site of intron 5, which codes for the phosphatase domain (Fig. 6).

**Table 1:** Mutations detected both in GCT and AML

Gene	Accession no.	Chr^a^	Exon	Nucleotide change	Amino acid change	Mutation type	VAF in GCT	VAF in AML
*ASF1A*	NM_014034	6	3	c.T231A	p.D77E	nonsynonymous SNV^b^	0.324	0.036
*DLG2*	NM_001142699	11	4	c.C140T	p.T47I	nonsynonymous SNV	0.468	0.215
*EDRF1*	NM_001202438	10	22	c.C3172T	p.H1058Y	nonsynonymous SNV	0.259	0.093
*GOLGA8J*	NM_001282472	15	12	c.G992A	p.R331H	nonsynonymous SNV	0.181	0.095
*PCLO*	NM_014510	7	13	c.T13947G	p.H4649Q	nonsynonymous SNV	0.243	0.119
*PTEN*	NM_000314	10	5	c.492 + 1G > A	p.V85Gfs*14	splicing	0.476	0.207
*RLF*	NM_012421	1	8	c.4563_4567del	p.I1521fs	frameshift deletion	0.357	0.218
*TP53*	NM_000546	17	8	c.G836A	p.G279E	nonsynonymous SNV	0.481	0.176
*YY2*	NM_206923	X	1	c.G813A	p.M271I	nonsynonymous SNV	0.614	0.176

Details of mutated genes in both germ cell tumor (GCT) and acute myeloid leukemia (AML). ^a^ *Chr* Chromosome; ^b^ *SNV* Single nucleotide variant

## Discussion

The prognosis of primary non-seminomatous mGCTs in the absence of HMs is poor with a 5-year overall survival (OS) of 45%, compared with that of ~ 90% in pure seminoma irrespective of the primary site [1]. In comparison, the prognosis of patients with mGCT and associated HM is extremely poor, with a median OS of 5 months [5]. This dismal prognosis held true in the current case. The standard chemotherapy for GCT had little effect in this case. The induction therapy for AML did not improve the mGCT, and it grew larger. The AML-associated thrombocytopenia made it difficult to perform chemotherapy for the mGCT.

Previous research demonstrating isochromosome 12p in both GCTs and HMs suggested that these malignancies had a common progenitor, and the identification of the same gene mutations, including of *TP53* and *PTEN,* in both mGCTs and AML samples in two cases established the idea that the mGCT and AML share a founding clone [6, 9, 10]. In the present case, the common cytogenetic abnormalities, namely trisomy 6, tetrasomy 8, trisomy 12, and trisomy 21, were detected in both tumors, although the detection method was different (G-banding analysis or FISH analysis; Figs. 3 and 4). WES analysis demonstrated 9 commonly mutated genes, including *TP53* and *PTEN* mutations, even though their contributions to the tumor genesis have not been elucidated. In addition, 9 other mutated genes were detected only in AML samples, while 7 other mutated genes occurred only in the GCT samples. These mutation profiles in AML and GCT strongly indicate that both originated from a common progenitor. The occurrence of 4 gene mutations in *PCLO*, *GOLGA8J, EDRF1,* and *ASF1A* on an initiator clone with *TP53, PTEN*, *RLF, DLG2,* and *YY2* mutations might have resulted in the establishment of the founder clone, which then developed separately along germ cell and hematopoietic lines by adding GCT- and AML-specific gene mutations, respectively. The progression of each tumor might have been mainly affected by its environment, and finally resulted in mGCT and AML, respectively. As mGCTs are cytogenetically identical to gonadal GCTs, they are thought to arise from the dissemination of early gonadal lesions [2]. The disseminated cells that recapitulate embryonal memory grow in the mediastinal region, and might develop into mGCTs. Hematopoietic cells traffic into and out of the thymus throughout postnatal and adult life via the thymic vasculature. The transforming cells with *TP53* and *PTEN* mutations in the mediastinal region might enter the BM, similar to homing of lymphoid cells.

Two cases harboring concurrent mutations of *TP53* and *PTEN* in both mGCTs and AMKL have been reported [9, 10], and our case is the third. As for the *TP53* mutation, a nonsynonymous mutation (exon2:c.389 T > C:p.L130P) and a frameshift mutation (exon10:c.7578213A > del:p.R213fs_del) in the DNA binding domain was reported in each case, which both lead to the loss of its transcription activity [9, 10, 12, 13]. In our case, similar to the previous two cases, the *TP53* mutation occurred in the DNA binding domain (exon8:c.G836A:p.G279E), and might cause the impairment of TP53 function (Fig. 6a). In case of the *PTEN* mutation, nonsynonymous mutations in phosphatase domain and C2 domain (exon6:c.725G > T:p.G251 V; exon10:c.89692922 T.C:p.C136R) were reported in each case [9, 10], which might lead to the reduction of PTEN’s membrane affinity, and subsequent loss of suppression of cell growth [14, 15]. In our case, the *PTEN* mutation occurred in the splicing donor site of intron 5 (exon5:c.492 + 1G > A), resulting in a PTEN splicing mutant (Fig. 6b) [16]. The same mutation has been reported in patents with Cowden syndrome, which causes hamartomatous neoplasms of the skin and mucosa, GI tract, CNS, and genitourinary tract, and an increased risk for malignancies of the breast, thyroid, and endometrium [16]. *TP53* mutations have been widely observed in a variety of tumors, including AML, but they are uncommon in GCT [17]. Similarly, *PTEN* mutations have been widely reported in many types of tumors. In HMs, *PTEN* deletions and mutations were detected in 10 and 27% of T-ALL cases, respectively, but the mutation is rare in AML [18]. Mice with heterozygous *PTEN* deletion demonstrated genomic instability and the development of multiple spontaneous tumors. The simultaneous depletion of TP53 and PTEN in mice promoted tumor genesis and metastasis [19], which might reflect the molecular pathology and the dismal prognosis of the concurrent disease of mGCT and AML.

In the concurrent cases of AML and mGCT, AML was diagnosed simultaneously at the diagnosis of mGCT or shortly after (occasionally < 6 months) starting the chemotherapy for mGCTs. In the latter cases, the chemotherapy for mGCT might accelerate the growth of AML cells and precipitate the onset of AML, because hematopoietic cells with *TP53* mutation are thought to grow dominantly compared with wild-type hematopoietic cells after chemotherapy, which is also speculated as a reason for secondary leukemia after chemotherapy [20, 21].

As for the treatment strategy, one report in which a 13-year-old boy was treated with AML regimens plus cisplatin, may be suggestive [22]. He undertook hematopoietic stem cell transplantation and surgical resection for AML and GCT, respectively, and survived. In addition to multipronged therapy, novel targeted therapies based on the molecular abnormalities may be required to improve the dismal prognosis [23–26].

## Conclusions

Considering both the dismal prognosis and the characteristic mutation profiles revealed by WES analysis, the associated mediastinal GCT and AMKL harboring *TP53* and *PTEN* mutations represent a unique biological entity.

## Data Availability

The datasets generated and/or analyzed during the current study are available in the Japanese Genotype-phenotype Archive (accession number JGAS00000000211).

## Abbreviations

AFP: Alpha-fetoprotein
AMKL: Acute megakaryoblastic leukemia
AML: Acute myeloid leukemia
BEP: Bleomycin, etoposide, and cisplatin
BM: Bone marrow
CNS: Central nervous system
COSMIC: Catalogue of Somatic Mutations in Cancer
CT: Computed tomography
ESP: Exome Sequencing Project
FISH: Fluorescence in situ hybridization
GCT: Germ cell tumor
GI: Gastrointestinal
HM: Hematological malignancies
IGV: Integrative Genomics Viewer
MDS: Myelodysplastic syndrome
mGCT: Mediastinal germ cell tumor
NCBI: National Center for Biotechnology Information
NHLBI: National Heart, Lung, and Blood Institute
OS: Overall survival
SNP: Single nucleotide polymorphism
SNV: Single nucleotide variant
T-ALL: T-cell acute lymphoblastic leukemia
TGO: Paclitaxel, gemcitabine, oxaliplatin
TIP: Paclitaxel, ifosfamide, and cisplatin
VAF: Variant allele frequencies
WES: Whole-exome sequencing
βhCG: Beta-human chorionic gonadotropin
